# Reliability of pre-operative diffusion tensor imaging parameter measurements of the cervical spine in patients with cervical spondylotic myelopathy

**DOI:** 10.1038/s41598-020-74624-6

**Published:** 2020-10-15

**Authors:** Eugene Lee, Joon Woo Lee, Yun Jung Bae, Hyo Jin Kim, Yusuhn Kang, Joong Mo Ahn

**Affiliations:** Department of Radiology, Seoul National University Bundang Hospital, 166, Gumi-ro, Bundang-gu, Seongnam-si, 463-707 Gyeonggi-do Korea

**Keywords:** Diseases, Medical research

## Abstract

The present study assessed test–retest and inter-observer reliability of diffusion tensor imaging (DTI) in cervical spondylotic myelopathy (CSM), as well as the agreement among measurement methods. A total 34 patients (12 men, 22 women; mean age, 58.7 [range 45–79] years) who underwent surgical decompression for CSM, with pre-operative DTI scans available, were retrospectively enrolled. Four observers independently measured fractional anisotropy (FA) values twice, using three different measurement methods. Test–retest and inter-observer reliability was assessed using intraclass correlation coefficients (ICCs). Overall, inter-observer agreements varied according to spinal cord level and the measurement methods used, and ranged from poor to excellent agreement (ICC = 0.374–0.821), with relatively less agreement for the sagittal region of interest (ROI) method. The radiology resident and neuro-radiologist group showed excellent test–retest reliability at almost every spinal cord level (ICC = 0.887–0.997), but inter-observer agreements varied from fair to good (ICC = 0.404–0.747). Despite excellent test–retest reliability of the ROI measurements, FA measurements in patients with CSM varied widely in terms of inter-observer reliability. Therefore, DTI parameter data should be interpreted carefully when applied clinically.

## Introduction

Diffusion tensor imaging (DTI) based on proton mobility evaluates the water diffusion properties. DTI provides diffusion characteristics that reflect the tissue integrity of the white matter fibers with fractional anisotropy (FA) which means the global anisotropy of the analyzed structures^1^ and apparent diffusion coefficient (ADC) value. Previous studies have revealed that DTI is a useful diagnostic MR technique for detecting subtle microstructural damage to the spinal cord that appears the absence of apparent abnormalities on conventional T_2_-weighted MR images^2–9^.

Cervical spondylotic myelopathy (CSM) is a chronic compressive spinal cord disorder caused by spondylosis or disc degeneration^2,10–12^. This disease affects a relatively large population and can induce irreversible neurological injury through progression of myelopathy. Therefore, accurate evaluation of disease severity and assessment of postoperative outcomes are important for the treatment of patients with CSM. Currently, the diagnosis and assessment of CSM are usually performed using a combination of clinical evaluation and MRI study. However, the basic limitation of conventional MRI is that these directly obtained findings do not correlate accurately with the patient’s symptoms or functional status in general and cannot predict the prognosis after surgical decompression.

Previously, Demir et al.^13^ reported that ADC values and diffusion tensor measurements showed better sensitivity than T_2_-weighted imaging for detecting myelopathy in CSM patients. Recently, several reports have suggested that DTI might be useful for evaluating the severity of myelopathy and predicting surgical outcomes in CSM^7,14–16^. However, contrary to DTI of the brain, DTI of the cervical spinal cord has basic technical difficulties, including large motion artifacts caused by swallowing, respiratory movement, and cerebrospinal fluid pulsation; susceptibility artifacts caused by abrupt changes in internal structures antero-posteriorly; and practical difficulty in measuring the exact region-of-interest (ROI) in the spinal cord. For DTI parameter assessment, the ROI must be placed manually, and possible measurement error is a serious concern. For clinical application of DTI in assessing CSM, the examination should have acceptable reliability and reproducibility.

Nevertheless, few studies have reported on DTI reliability in patients with CSM. Therefore, this study assessed the test–retest and inter-observer reliability of cervical spinal cord DTI in CSM patients, as well as the agreement among three ROI measurement methods.

## Results

### Overall ICC values

ICC values for FA measurements at each spinal cord level among the four observers are shown in Table 1. The overall agreements among the four observers varied according to spinal cord level, and the assessed measurement methods ranged from having poor to having excellent agreement (ICC = 0.374–0.821). Only levels C3/C4 and C4/C5 showed good-to-excellent agreement among observers for the first and second measurements in both the mean and manual ROI methods (ICC = 0.732–0.821).

**Table 1:** Overall ICCs of FA measurements among the four observers.

Level	Measurement method	Observer 1/2/3/4 (1^st^ measurement) ICC(95% CI)	Observer 1/2/3/4(2^nd^ measurement) ICC(95% CI)
C1/2	Mean ROI	0.686 (0.543–0.808)	0.460 (0.287–0.639)
	Manual ROI	0.572 (0.408–0.727)	0.496 (0.324–0.668)
	Sagittal ROI	0.500 (0.383–0.710)	0.550 (0.383–0.710)
C2/3	Mean ROI	0.716 (0.581–0.828)	0.717 (0.583–0.829)
	Manual ROI	0.783 (0.670–0.872)	0.679 (0.535–0.803)
	Sagittal ROI	0.389 (0.216–0.579)	0.374 (0.201–0.565)
C3/4	Mean ROI	0.797 (0.690–0.881)	0.732 (0.602–0.839)
	Manual ROI	0.808 (0.705–0.888)	0.819 (0.720–0.894)
	Sagittal ROI	0.700 (0.562–0.818)	0.704 (0.566–0.820)
C4/5	Mean ROI	0.800 (0.693–0.883)	0.821 (0.723–0.896)
	Manual ROI	0.793 (0.684–0.879)	0.784 (0.671–0.873)
	Sagittal ROI	0.628 (0.473–0.767)	0.594 (0.433–0.743)
C5/6	Mean ROI	0.722 (0.589–0.832)	0.574 (0.411–0.728)
	Manual ROI	0.616 (0.459–0.759)	0.615 (0.457–0.758)
	Sagittal ROI	0.432 (0.259–0.616)	0.398 (0.224–0.586)
C6/7	Mean ROI	0.538 (0.370–0.701)	0.652 (0.502–0.785)
	Manual ROI	0.529 (0.360–0.694)	0.536 (0.367–0.699)
	Sagittal ROI	0.548 (0.381–0.709)	0.553 (0.386–0.712)
C7/T1	Mean ROI	0.726 (0.595–0.835)	0.657 (0.508–0.788)
	Manual ROI	0.650 (0.500–0.783)	0.618 (0.461–0.760)
	Sagittal ROI	0.430 (0.256–0.613)	0.404 (0.230–0.592)

Two elective medical university students (observers 1 and 2), one third-year radiology resident (observer 3), and one neuro-radiologist with 2 years of experience in diffusion tensor imaging (observer 4).

FA, fractional anisotropy, ICC, intraclass correlation coefficient, CI, confidence interval, ROI, region of interest.

Among the methods, sagittal ROI measurements showed relatively lower agreement among observers for the C6/C7 level than almost all spinal cord levels, except for the second measurement of C1/C2. Moreover, lower ICC values, reflecting poor inter-observer agreement, were found at the C2/C3, C5/C6, and C7/T1 levels using the sagittal ROI method (ICC = 0.374–0.432).

### Test–retest and inter-observer reliability

ICC values for the four observers from the test–retest reliability assessment are demonstrated in Table 2. Test–retest reliability varied among observers 1 and 2 (ICC = 0.460–0.959); however, observers 3 and 4 showed excellent test–retest reliability at all spinal cord levels for the three measurement methods (ICC = 0.887–0.997), except for observer 4 at the C1/C2 level using the sagittal ROI method (ICC = 0.645).

**Table 2:** Test–retest reliability of the three measurement methods among the four observers.

Level	Measurement method	Observer 1 ICC(95% CI)	Observer 2 ICC(95% CI)	Observer 3 ICC(95% CI)	Observer 4 ICC(95% CI)
C1/2	Mean ROI	0.460 (0.149–0.688)	0.558 (0.275–0.752)	0.986 (0.973–0.993)	0.995 (0.990–0.997)
	Manual ROI	0.541 (0.253–0.741)	0.524 (0.230–0.730)	0.969 (0.940–0.985)	0.997 (0.9932–0.998)
	Sagittal ROI	0.810 (0.653–0.901)	0.684 (0.453–0.828)	0.889 (0.790–0.943)	0.645 (0.396–0.805)
C2/3	Mean ROI	0.892 (0.794–0.944)	0.919 (0.845–0.959)	0.977 (0.955–0.987)	0.992 (0.985–0.996)
	Manual ROI	0.798 (0.632–0.894)	0.888 (0.788–0.943)	0.944 (0.891–0.972)	0.993 (0.986–0.996)
	Sagittal ROI	0.646 (0.398–0.806)	0.816 (0.663–0.904)	0.926 (0.856–0.962)	0.973 (0.948–0.987)
C3/4	Mean ROI	0.948 (0.898–0.974)	0.843 (0.709–0.919)	0.991 (0.982–0.996)	0.992 (0.983–0.996)
	Manual ROI	0.914 (0.834–0.956)	0.868 (0.753–0.932)	0.989 (0.978–0.995)	0.984 (0.968–0.992)
	Sagittal ROI	0.891 (0.794–0.944)	0.942 (0.886–0.970)	0.913 (0.834–0.956)	0.965 (0.931–0.982)
C4/5	Mean ROI	0.910 (0.828–0.954)	0.959 (0.920–0.979)	0.945 (0.894–0.972)	0.994 (0.989–0.997)
	Manual ROI	0.923 (0.851–0.961)	0.917 (0.840–0.957)	0.984 (0.968–0.992)	0.990 (0.980–0.995)
	Sagittal ROI	0.797 (0.631–0.893)	0.808 (0.650–0.899)	0.865 (0.746–0.930)	0.974 (0.949–0.987)
C5/6	Mean ROI	0.635 (0.383–0.800)	0.807 (0.647–0.899)	0.964 (0.930–0.982)	0.980 (0.961–0.990)
	Manual ROI	0.888 (0.788–0.943)	0.783 (0.607–0.885)	0.981 (0.963–0.991)	0.994 (0.988–0.997)
	Sagittal ROI	0.659 (0.418–0.814)	0.840 (0.703–0.917)	0.934 (0.872–0.966)	0.887 (0.785–0.942)
C6/7	Mean ROI	0.582 (0.308–0.767)	0.813 (0.657–0.902)	0.946 (0.895–0.973)	0.990 (0.980–0.995)
	Manual ROI	0.660 (0.418–0.814)	0.683 (0.452–0.828)	0.966 (0.932–0.983)	0.992 (0.984–0.996)
	Sagittal ROI	0.518 (0.223–0.727)	0.813 (0.658–0.902)	0.924 (0.854–0.961)	0.977 (0.955–0.989)
C7/T1	Mean ROI	0.834 (0.692–0.913)	0.735 (0.532–0.858)	0.980 (0.961–0.990)	0.995 (0.989–0.997)
	Manual ROI	0.814 (0.659–0.903)	0.711 (0.494–0.844)	0.972 (0.944–0.986)	0.983 (0.966–0.992)
	Sagittal ROI	0.798 (0.632–0.894)	0.815 (0.661–0.903)	0.919 (0.844–0.959)	0.924 (0.854–0.961)

Two elective medical university students (observers 1 and 2), one third-year radiology resident (observer 3), and one neuro-radiologist with 2 years of experience in diffusion tensor imaging (observer 4).

FA, fractional anisotropy, ICC, intraclass correlation coefficient, CI, confidence interval, ROI, region of interest.

Based on the test–retest reliability results, the four observers were divided into two groups: the medical student group (observers 1 and 2) and the radiology resident and neuro-radiologist group (observers 3 and 4). ICC values within these two groups for the three measurement methods are shown in Table 3. Inter-observer agreement between observers 1 and 2 varied widely across the different spinal cord levels and measurement methods (ICC = 0.510–0.954), which were similar to the test–retest reliability results.

**Table 3:** Inter-observer reliability for the three measurement methods within two groups.

Level	Measurement Method	Observer 1/2*		Observer 3/4†	
		1st measurement ICC(95% CI)	2nd measurement ICC(95% CI)	1st measurement ICC(95% CI)	2nd measurement ICC(95% CI)
C1/2	Mean ROI	0.775 (0.595–0.881)	0.510 (0.213–0.721)	0.690 (0.462–0.832)	0.647 (0.400–0.807)
	Manual ROI	0.744 (0.546–0.864)	0.635 (0.382–0.799)	0.551 (0.266–0.747)	0.579 (0.304–0.765)
	Sagittal ROI	0.809 (0.651–0.900)	0.781 (0.604–0.884)	0.479 (0.173–0.701)	0.430 (0.113–0.668)
C2/3	Mean ROI	0.925 (0.855–0.962)	0.906 (0.819–0.952)	0.652 (0.406–0.810)	0.700 (0.477–0.838)
	Manual ROI	0.930 (0.864–0.964)	0.789 (0.618–0.889)	0.747 (0.551–0.865)	0.709 (0.491–0.843)
	Sagittal ROI	0.667 (0.428–0.818)	0.814 (0.660–0.903)	0.275 (-0.065–0.558)	0.302 (-0.036–0.578)
C3/4	Mean ROI	0.954 (0.909–0.977)	0.868 (0.752–0.932)	0.682 (0.451–0.827)	0.646 (0.398–0.806)
	Manual ROI	0.899 (0.807–0.948)	0.923 (0.851–0.961)	0.792 (0.622–0.890)	0.800 (0.636–0.895)
	Sagittal ROI	0.924 (0.853–0.961)	0.907 (0.823–0.953)	0.532 (0.241–0.735)	0.624 (0.366–0.793)
C4/5	Mean ROI	0.948 (0.898–0.974)	0.932 (0.869–0.966)	0.663 (0.423–0.816)	0.757 (0.566–0.871)
	Manual ROI	0.967 (0.936–0.984)	0.913 (0.834–0.956)	0.756 (0.565–0.870)	0.773 (0.591–0.880)
	Sagittal ROI	0.795 (0.628–0.892)	0.830 (0.689–0.912)	0.422 (0.102–0.662)	0.410 (0.088–0.654)
C5/6	Mean ROI	0.783 (0.608–0.885)	0.580 (0.305–0.765)	0.641 (0.391–0.803)	0.634 (0.380–0.799)
	Manual ROI	0.843 (0.708–0.918)	0.838 (0.700–0.916)	0.594 (0.325–0.774)	0.607 (0.342–0.782)
	Sagittal ROI	0.676 (0.442–0.824)	0.771 (0.589–0.879)	0.222 (-0.121–0.517)	0.404 (0.081–0.650)
C6/7	Mean ROI	0.596 (0.327–0.776)	0.854 (0.727–0.924)	0.461 (0.150–0.689)	0.555 (0.271–0.750)
	Manual ROI	0.728 (0.520–0.854)	0.617 (0.357–0.789)	0.478 (0.1716–0.700)	0.570 (0.291–0.759)
	Sagittal ROI	0.649 (0.402–0.808)	0.780 (0.604–0.884)	0.595 (0.326–0.775)	0.628 (0.372–0.795)
C7/T1	Mean ROI	0.892 (0.795–0.945)	0.675 (0.440–0.823)	0.678 (0.445–0.825)	0.694 (0.469–0.834)
	Manual ROI	0.861 (0.740–0.928)	0.799 (0.635–0.894)	0.715 (0.501–0.847)	0.679 (0.447–0.826)
	Sagittal ROI	0.762 (0.575–0.874)	0.764 (0.578–0.875)	0.084 (-0.257–0.406)	0.157 (-0.186–0.467)

*The group with two medical students (observers 1 and 2).

^†^The group with a radiology resident and neuro-radiologist (observers 3 and 4).

FA, fractional anisotropy, ICC, intraclass correlation coefficient, CI, confidence interval, ROI, region of interest.

However, despite the excellent test–retest reliability, the inter-observer agreements of the radiology resident and neuro-radiologist group (observers 3 and 4) also varied widely, with fair-to-good agreement (ICC = 0.404–0.747) for almost every spinal segment and all three measurement methods. In particular, there was poor inter-observer agreement at the C2/C3 (ICC = 0.275 and 0.302 for the first and second measurements, respectively), C5/C6 (ICC = 0.222 and 0.404 for the first and second measurements, respectively), and C7/T1 levels (ICC = 0.084 and 0.157 for the first and second measurements, respectively) when using the sagittal ROI method. There was excellent inter-observer agreement only at the C3/C4 (ICC = 0.792 and 0.800 for the first and second measurements, respectively) and C4/C5 levels (ICC = 0.756 and 0.773 for the first and second measurements, respectively) when using the manual ROI method.

The differences between observers 3 and 4 in the calculated mean FA values from C1/C2 through C7/T1 for all subjects are shown in Table 4. Between observers 3 and 4, there were statistically significant differences in mean FA values for all three measurement methods. Among the three methods, the sagittal ROI method yielded a statistically significantly lower value than did the other methods for both observers. There was no statistically significant difference in the mean FA values obtained by the mean versus manual ROI measurements for both observers.

**Table 4:** Differences in mean FA values from C1/2 through C7/T1 for all subjects between observers 3 and 4.

Measurement method	Observer 3*	Observer 4†	P value
Mean ROI	0.617 ± 0.077‡	0.599 ± 0.072	0.015
Manual ROI	0.616 ± 0.080	0.597 ± 0.070	0.006
Sagittal ROI	0.549 ± 0.083	0.579 ± 0.074	0.020

*Third-year radiology resident (observer 3).

^†^Neuro-radiologist with 2 years of experience in diffusion tensor imaging (observer 4).

^‡^Mean FA value ± standard deviation.

FA, fractional anisotropy, ROI, region of interest.

## Discussion

This study is one of the first to evaluate the reliability of DTI in assessing the cervical spinal cord in adult patients with CSM. The aim was to assess the test–retest and inter-observer reliability of FA values at all intervertebral disc levels of the whole cervical spinal cord in patients with CSM. To date, only three studies^17–19^ have reported assessment of the reliability of ROI placement to quantify DTI measurements in the cervical spinal cord. Two studies were performed on pediatric spinal cords, with or without spinal cord injury. Only one study was performed on the cervical spinal cord in a healthy adult population.

Mulcahey et al.^18^ reported good to strong test–retest reliability for diffusivity values at each level of the spinal cord. Likewise, the reliability of FA values for the mid-C4 level and levels at and below C5–C6 was good. Despite only fair repeatability for FA values at several levels, the data suggested that repeated DTI values can be obtained for children with chronic cervical-level spinal cord injury, with evidence of good to strong reliability for mean diffusivity (MD), axial diffusivity (AD), and radial diffusivity (RD), and fair-to-good reliability for FA.

Barakat et al.^17^ also reported that inter- and intra-observer agreement between two ROI measurement methods (a freehand ROI and a fixed-size ROI) showed moderate (ICC = 0.5) to strong (ICC = 0.84) agreement in the normal pediatric spinal cord, and that FA values showed the highest variability among DTI parameters (ICC = 0.10–0.87).

Brander et al.^19^ conducted a study on the reproducibility of ROI measurements using a freehand technique in a healthy adult population. They reported that the intra-observer variation of the measurements for whole-cord FA and for the ADC showed almost perfect agreement, when using both ROI- and tractography-based measurements. There was greater variation in measurements of individual columns. Inter-observer agreement varied from moderate to strong for whole-cord FA and for the ADC.

**Figure 1:**
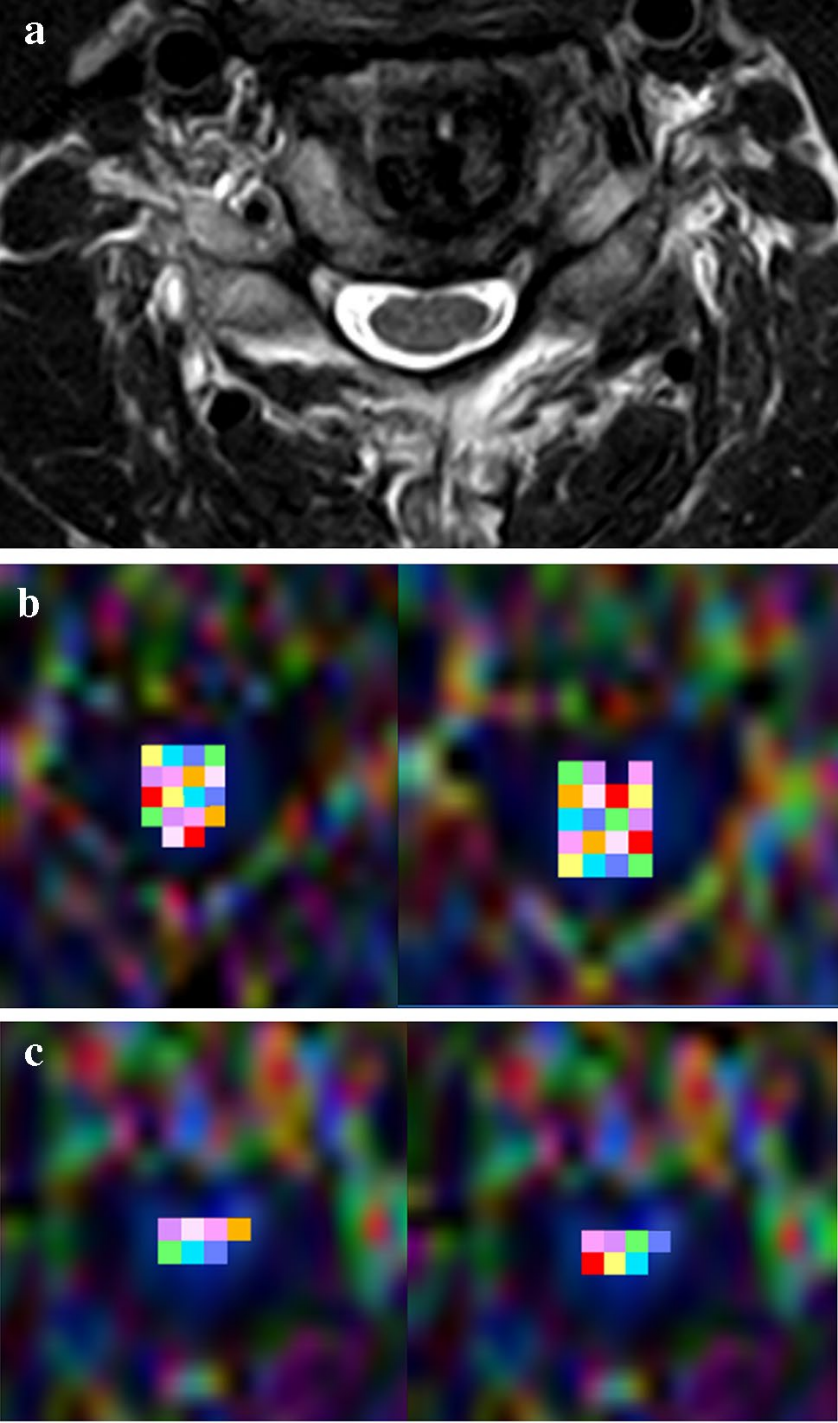
Fractional anisotropy (FA) measurements by observers 1 and 3 at the C5/C6 level using the mean region-of-interest (ROI) method in diffusion tensor images of a 64-year-old man. (**a**) An axial T_2_-weighted turbo spin-echo (TSE) scan of the C5/C6 intervertebral disc level is shown. The spinal cord and adjacent cerebrospinal fluid (CSF) space are clearly demonstrated. (**b**) The first and second FA measurements by observer 1 at the C5/C6 level obtained using the mean ROI method. The calculated mean FA values are 0.424 and 0.400. (**c**) The first and second FA measurements by observer 3 at the C5/C6 level obtained using the mean ROI method. The calculated mean FA values are 0.635 and 0.635.

In our study, repeated ROI measurements revealed wide variations in the inter-observer reliability, which was in contrast to the findings of previous articles. Although the radiology resident and neuro-radiologist group (observers 3 and 4) showed excellent test–retest reliability, inter-observer reliability showed fair-to-good agreement for almost every spinal segment using the three measurement methods. Furthermore, there were statistically significant differences in the calculated mean FA values obtained when these observers used the three ROI measurement methods. This finding has practical implications because DTI data for the analysis of cervical cord abnormalities can include a basic measurement error bias, which may cause a reliability problem in clinical use. If the placement of the ROI includes more CSF or adjacent structures, the FA value will decrease; Fig. 1 clearly demonstrates this phenomenon. Observer 1 placed more single-voxel ROIs than did observer 3. According to the axial T_2_-weighted TSE scan, the coverage of the total ROI placement exceeded the true outline of the spinal cord and contained more CSF. Consequently, calculated FA values were markedly lower for observer 1 than for observer 3. Furthermore, in patients with CSM, a compressed spinal cord and the low spatial resolution of DTI make it difficult to place an exact ROI that includes only the spinal cord. Recently, several reports have indicated that pre-operative FA values correlated well with symptoms or functional status in patients with CSM and could predict surgical outcomes^7,14,15^. According to our results, DTI parameters and clinical data analysis should be interpreted with caution.

According to our data, the sagittal ROI measurement method showed lower test–retest and inter-observer reliability and statistically different mean FA values as compared with the other methods. Thus, it seems that the sagittal ROI method is not appropriate for measurement in a clinical setting. Particularly at the C2/C3, C5/C6, and C7/T1 levels, there was poor inter-observer agreement when using sagittal ROI measurement. This result is similar to that of Barakat et al.^17^. The upper cervical (C2/C3) and lower cervical (C7/T1) levels were located at the edge of the coil that was used for imaging the patients. In these regions, the signal-to-noise ratio (SNR) decreases and the signal drop is marked. Furthermore, the relatively wide CSF space, as compared to other spinal levels, may lead to inclusion of more CSF when placing the ROI. All these factors can contribute to lower inter-observer agreement when using the sagittal ROI method. The low agreement at the C5/C6 level may have been related to the C5/C6 level being the most commonly affected segment, with central canal compromise, in patients with CSM, while the lowest cervical levels (C4–C7) were most sensitive to cardiac motion. Therefore, obscured anatomical margins and some cardiac-related artifacts may have biased the placement of ROIs.

However, about the resolution against this ROI problems, Yokohama et al.^20^ reported the more reliable and better visibility DTI method in 3-T MRI, called reduced FOV or so-called zonally oblique multislice (ZOOM) DTI. They concluded that ZOOM DTI provides better visibility with less distortion and high accuracy using a small FOV and a shorter practical scan time compared with conventional DTI. Moreover, using this ZOOM DTI method, Iwasaki et al.^21^ reported the pre-surgical FA values are affected by "aligend fibers effect", which is compressed fibers show higher FA value and those values are not suitable for prognostic predictors. After all, it is thought that accurate parameter measurement is important for DTI's clinical utility and therefore technical improvement is necessary to clearly distinguish the boundaries between the spinal cord and CSF by reducing image distortion and improving the spatial resolution. A method like ZOOM DTI mentioned above would be a good solution and authors also proposed that to attain further rapid, high resolution DTI sequences, combined ZOOM DTI and recently introduced techniques such as turbo spin-echo (TSE)-DWI and multi-band SENSE are desirable.

There are some limitations to our study. First, the resolution of the DTI in this study is low, and this may have increased the deviation between inter-observers in accessing the reliability. In fact, our institution currently uses DTI protocol by increasing slice thickness and NEX (to 4 mm and 14 respectively), and the reduced FOV technology could also be a solution to increase resolution. If there was a sufficient increase in resolution, it is possible that the reliability of the DTI parameter measurements has increased. In addition, appropriate anatomic reference may have to be provided, but only in the sagittal ROI measurement method used the guidance of the T2-weighted scan. If T2-based (T2WI or T2* weighted image) references were used, it would yield better reproducibility of placement of ROI. Second, only patients with CSM were enrolled. Compared with a normal healthy population, anatomical changes, such as underlying spondylosis or cord compression, may make it difficult to place the ROI exactly on the spinal cord. However, CSM is the most common form of spinal cord dysfunction^22^ and the reliability of FA measurements in this group of patients has clinical importance. Third, although all observers who performed measurements had a consensus training session for ROI placement before the study, their experience in DTI and related measurements was relatively low. Furthermore, the training using the standardized protocol was insufficient, especially in the unexperienced observer, it was possible that these problems were flawed in interpreting the results of the reliability. The result that test–retest values are high and inter-observer reliability is relatively low itself can be said to mean the fact that each observer has applied different measurement methods depending on the understanding of anatomy and MR images, even though the consensus training was performed prior to the measurement. Therefore, it is believed that the reliability of DTI can be concluded in a true sense if the training using standardized protocol is conducted prior to the study, and when experts with sufficient practical experience evaluate as observers. Fourth, we did not use cardiac gating during the image acquisition, which can diminish flow artifacts from CSF. However, using cardiac gating may increase other motion artifacts caused by respiration or swallowing, because of the lengthened examination time. Fifth, we evaluated only FA values among DTI parameters, and did not investigate the ADC, AD, RD, or MD. Previous studies^17,18^ demonstrated relatively low and variable reliability in FA values as compared with other diffusion parameters. Thus, further study evaluating the reliability of other DTI parameters is needed to reveal overall reliability. Finally, the ideal reliability study requires a prospective study design using a priori hypothesis and a positive design, high resolution DTI study is required to support our findings.

In conclusion, for use as a diagnostic tool, data obtained by measurements on DTI should have high reliability and reproducibility. Despite excellent test–retest reliability of the ROI measurements, FA values in patients with CSM varied widely in terms of inter-observer reliability in our study. Therefore, DTI parameters should be interpreted with caution when applied clinically. Furthermore, education and practical training in DTI methods are imperative to ensure for reliable assessment for the measurements.

## Methods

This retrospective study was approved by the Seoul National University Bundang Hospital institutional review board (IRB No: B-1406-256-102). This study was conducted in accordance with relevant guidelines and regulations/declaration of Helsinki. The research holds out no more than minimal risks to participants and was reviewed through an Expedited Review. So, the requirement for informed consent was waived by the Seoul National University Bundang Hospital institutional review board.

### Study subjects

We retrospectively searched the electronic medical record system and Radiology Department database at our institution between July 2013 and December 2013, for cases meeting the following inclusion criteria: (1) a clinical diagnosis of CSM, (2) availability of pre-operative diffusion tensor MRI scans, and (3) the use of surgical decompression. All patients had neurological signs and symptoms with clear evidence of cervical spinal cord compression due to cervical spondylosis on conventional cervical spine MRI. The evaluation of myelopathy was performed using a modified Japanese Orthopedic Association score. We excluded patients with (1) tumor-, trauma-, or infection-related cord compression, (2) prior surgery, (3) coexisting neurologic disorders, such as acute transverse myelitis or multiple sclerosis, and (4) suboptimal image quality due to severe artifacts.

Finally, 34 patients (12 men, 22 women; mean age, 58.7 [range 45–79] years) were enrolled in this study. For surgical treatment, anterior cervical discectomy and fusion was performed in 28 cases, laminoplasty was performed in four cases, and posterolateral fusion was performed in two cases.

### DTI protocol

All pre-operative DTI was performed within 2 weeks prior to surgery. All MRI examinations of the cervical spinal cord were performed using a 3-T MRI scanner (Achieva, Philips Medical Systems, Best, The Netherlands). No upgrade or other changes were made to the MRI system software in this study. During the image acquisition process, all subjects were placed in the supine position with 16-channel neurovascular coils applied to the cervical region.

Sensitivity-encoding (SENSE) single-shot echo planar imaging (EPI) was used. Used MR protocols are^23^:

1. SENSE factor: 4 (for sagittal DTI). excitations: 4
2. b-value: 600 s/mm^2^
3. diffusion gradient directions: 15, diffusion gradient strength: 40 mT/m
4. slice thickness: 2 mm
5. fold-over direction: anterior–posterior, fat shift direction: posterior
6. TR/TE: 3,400/60 ms, matrix: 124 × 124 mm, FOV: 250 mm, Voxel size: 2 × 2 × 2 mm
7. Scan time: 4 min

After sending all source images of the DTI to a personal computer, diffusion tensor parameters/fiber tracking were evaluated using the fiber assignment by continuous tracking (FACT) algorithm implemented within the DTI task card software (the Extended MR WorkSpace 2.6, Philips Medical Systems)^6,24^. In the axial b0 image, two slices (C1 and C7 levels) were selected. Circular ROI that included the entire spinal cord was placed and fiber tracking was performed. Only fibers passing through the ROIs were displayed. The thresholds for tracking termination were 0.2 for FA and 30° for the angle between 2 contiguous eigen-vectors.

### Image and measurement analysis

A total of four observers (two elective medical university students [observers 1 and 2], one third-year radiology resident [observer 3], and one neuro-radiologist with 3 years of experience in DTI [observer 4]) independently measured FA values, twice, after consensus training. To prevent recall bias, each measurement was performed at an interval of 1 month. After sending all source DTI images to a personal computer, each observer, who was blinded to the clinical condition of each patient, measured the FA value in the cervical spinal cord at the level of each spine segment. For the FA measurements, ROIs were manually drawn on axial and sagittal color tensor maps along the cervical spinal cord at the level of each cervical intervertebral disc. Spine segments were selected for each disc level from C1/2 to C7/T1, with reference to a mid-sagittal T_1_-weighted image.

**Figure 2:**
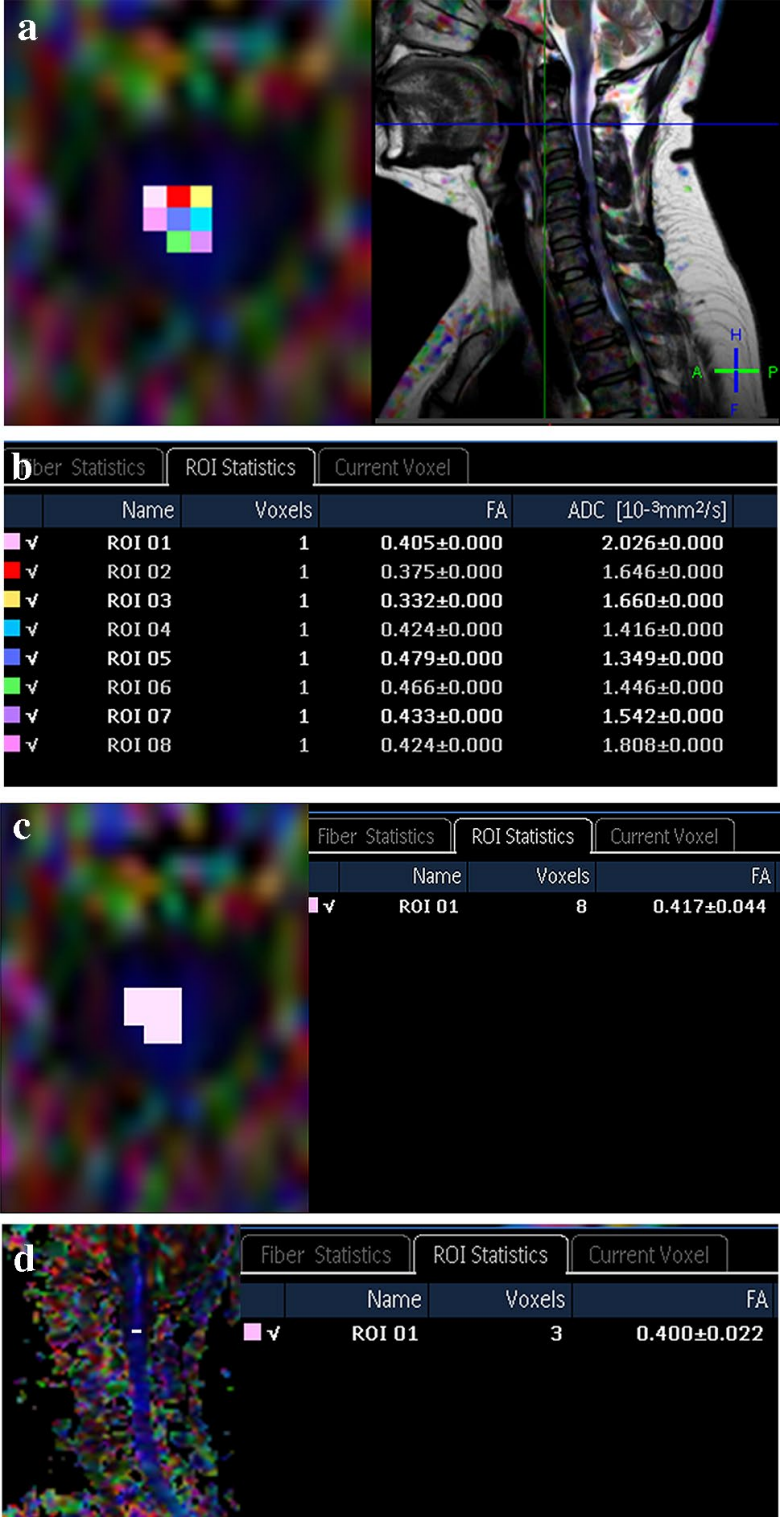
Three different fractional anisotropy (FA) measurement methods at the C2/C3 level applied in diffusion tensor images of a 53-year-old woman. (**a**) The mean region-of-interest (ROI) method using each voxel inside the spinal cord on the axial image, guided by a sagittal T_2_-weighted turbo spin-echo (TSE) image. A total of eight voxels were placed on the spinal cord. (**b**) The calculated average FA values for all voxels inside the spinal cord from the mean ROI method range from 0.332 to 0.479. These FA values were averaged per cord level across all subjects. In this patient, the mean FA value is 0.417. Additional apparent diffusion coefficient (ADC) values for each voxel were also automatically calculated. (**c**) The manual ROI method, using a freehand technique, which represents approximately one voxel. The calculated FA value is 0.417. (**d**) The sagittal ROI method, using a freehand technique, which represents approximately one voxel. The calculated FA value is 0.400.

Three measurement methods were used for placing the ROIs in this study. (1) In the mean ROI method, for each single voxel inside the spinal cord on the axial image, special attention was paid to select ROIs while avoiding partial volume effects, magnetic susceptibility effects, and motion artifacts. Average FA values for all voxels inside the spinal cord at each spine segment level were calculated (Fig. 2a,b). (2) In the manual ROI method, each observer manually outlined an ROI up to the outer margin of the spinal cord on an axial FA map, using a freehand technique, which represented approximately one voxel, while being cautious to avoid volume-averaging effects with the cerebrospinal fluid (CSF) (Fig. 2c). (3) In the sagittal ROI method, each ROI was placed manually on the sagittal FA map, similar to the second method (manual ROI) (Fig. 2d). In this method, ROI selection for each spinal level was guided by reconstructed sagittal b0 maps, and axial and sagittal turbo spin-echo (TSE) T_2_-weighted images.

One of the authors (musculoskeletal radiologist with 4 years of experience in spinal DTI analysis) conducted the image and measurement analyses. To assess test–retest and inter-observer reliability, the FA values measured by the four observers using these three methods were compared.

### Statistical analysis

Statistical analyses were performed by one author. The test–retest- and inter-observer reliability of each FA value obtained by the four observers using three measurement methods were assessed using intraclass correlation coefficients (ICCs) and a two-way random model. Test–retest and inter-observer reliability depends primarily on good training of the observers and good standardization of the task. The ICC value could range from 0 to 1; ICC values of less than 0.40 represented poor agreement, values of 0.40–0.75 represented fair-to-good agreement, and values greater than 0.75 represented excellent agreement.

The differences in the mean FA value, averaged per cord level from C1/C2 through C7/T1 of all study subjects, for all three measurement methods among the observers were assessed using the wilcoxon signed rank test test. Analyses were performed using SPSS (ver. 21.0, SPSS Inc., Chicago, IL, USA) and MedCalc software (version 13.0, MedCalc Software, Mariakerke, Belgium). A P value < 0.05 was considered statistically significant.

## Data Availability

The datasets generated during and/or analyzed during the current study are available from the corresponding author on reasonable request.

## References

[1] Le Bihan D (2003). Looking into the functional architecture of the brain with diffusion MRI. Nat. Rev. Neurosci..

[2] McCormick WE, Steinmetz MP, Benzel EC (2003). Cervical spondylotic myelopathy: make the difficult diagnosis, then refer for surgery. Cleve. Clin. J. Med..

[3] Ducreux D (2007). Diffusion tensor magnetic resonance imaging and fiber tracking in spinal cord lesions: current and future indications. Neuroimaging Clin. N. Am..

[4] Clark CA, Werring DJ (2002). Diffusion tensor imaging in spinal cord: methods and applications—a review. NMR Biomed..

[5] Facon D (2005). MR diffusion tensor imaging and fiber tracking in spinal cord compression. AJNR. Am. J. Neuroradiol..

[6] Lee JW (2011). Diffusion tensor imaging and fiber tractography in cervical compressive myelopathy: preliminary results. Skeletal Radiol..

[7] Wen CY (2014). Is diffusion anisotropy a biomarker for disease severity and surgical prognosis of cervical spondylotic myelopathy?. Radiology.

[8] Van Hecke W (2009). A diffusion tensor imaging group study of the spinal cord in multiple sclerosis patients with and without T2 spinal cord lesions. J. Mag. Reson..

[9] Werring D, Clark C, Barker G, Thompson A, Miller D (1999). Diffusion tensor imaging of lesions and normal-appearing white matter in multiple sclerosis. Neurology.

[10] Ichihara K, Taguchi T, Sakuramoto I, Kawano S, Kawai S (2003). Mechanism of the spinal cord injury and the cervical spondylotic myelopathy: new approach based on the mechanical features of the spinal cord white and gray matter. J. Neurosurg..

[11] Rao R (2002). Neck pain, cervical radiculopathy, and cervical myelopathy. J. Bone Joint Surg..

[12] Bernhardt M, Hynes R, Blume H, White A (1993). Cervical spondylotic myelopathy. J. Bone Joint Surg..

[13] Demir A (2003). Diffusion-weighted MR imaging with apparent diffusion coefficient and apparent diffusion tensor maps in cervical spondylotic myelopathy. Radiology.

[14] Rajasekaran S (2014). The assessment of neuronal status in normal and cervical spondylotic myelopathy using diffusion tensor imaging. Spine.

[15] Jones J, Cen S, Lebel R, Hsieh P, Law M (2013). Diffusion tensor imaging correlates with the clinical assessment of disease severity in cervical spondylotic myelopathy and predicts outcome following surgery. AJNR. Am. J. Neuroradiol..

[16] Kerkovský M (2012). Magnetic resonance diffusion tensor imaging in patients with cervical spondylotic spinal cord compression: correlations between clinical and electrophysiological findings. Spine.

[17] Barakat N (2015). Inter-and intra-rater reliability of diffusion tensor imaging parameters in the normal pediatric spinal cord. World J. Radiol..

[18] Mulcahey M (2012). Diffusion tensor imaging in pediatric spinal cord injury: preliminary examination of reliability and clinical correlation. Spine.

[19] Brander A (2014). Diffusion tensor imaging of the cervical spinal cord in healthy adult population: normative values and measurement reproducibility at 3T MRI. Acta. Radiol..

[20] Yokohama T (2019). The reliability of reduced field-of-view DTI for highly accurate quantitative assessment of cervical spinal cord tracts. Magn. Reson. Med. Sci..

[21] Iwasaki M (2019). Decreased value of highly accurate fractional anisotropy using 3-tesla ZOOM diffusion tensor imaging after decompressive surgery in patients with cervical spondylotic myelopathy: aligned fibers effect. World Neurosurg. X..

[22] Montgomery D, Brower R (1992). Cervical spondylotic myelopathy. Clinical syndrome and natural history. Orthop. Clin. N. Am..

[23] Budzik J-F, Balbi V, Le Thuc V, Duhamel A, Assaker R, Cotten A (2011). Diffusion tensor imaging and fibre tracking in cervical spondylotic myelopathy. Eur. Radiol..

[24] Aota Y (2008). The correlation of diffusion-weighted magnetic resonance imaging in cervical compression myelopathy with neurologic and radiologic severity. Spine.

